# Rehabilitation using virtual gaming for Hospital and hOMe-Based training for the Upper limb in acute and subacute Stroke (RHOMBUS II): results of a feasibility randomised controlled trial

**DOI:** 10.1136/bmjopen-2024-089672

**Published:** 2025-01-28

**Authors:** Tom Butcher, Alyson Warland, Victoria Stewart, Basaam Aweid, Arul Samiyappan, Elmar Kal, Jennifer Ryan, Dimitrios A Athanasiou, Karen Baker, Guillem Singla-Buxarrais, Nana Anokye, Carole Pound, Francesca Gowing, Meriel Norris, Cherry Kilbride

**Affiliations:** 1 Department of Sport, Exercise and Rehabilitation, Northumbria University, Newcastle upon Tyne, UK; 2 Department of Health Sciences, Brunel University of London, Uxbridge, UK; 3 Hillingdon Hospitals NHS Foundation Trust, Uxbridge, UK; 4 Central and North West London NHS Foundation Trust, London, UK; 5 School of Physiotherapy, Royal College of Surgeons in Ireland, Dublin, Ireland; 6 Neurofenix, London, UK; 7 Neurofenix, Atlanta, Georgia, USA; 8 Independent Researcher, London, UK

**Keywords:** REHABILITATION MEDICINE, Stroke, Telemedicine

## Abstract

**Objective:**

To investigate the safety, feasibility and acceptability of the Neurofenix platform for upper-limb rehabilitation in acute and subacute stroke.

**Design:**

A feasibility randomised controlled trial with a parallel process evaluation.

**Setting:**

Acute Stroke Unit and participants’ homes (London, UK).

**Participants:**

24 adults (>18 years), acute and subacute poststroke, new unilateral weakness, scoring 9–25 on the Motricity Index (elbow and shoulder), with sufficient cognitive and communicative abilities to participate.

**Interventions:**

Participants randomised to the intervention or control group on a 2:1 ratio. The intervention group (n=16) received usual care plus the Neurofenix platform for 7 weeks. The control group (n=8) received usual care only.

**Outcomes:**

Safety was assessed through adverse events (AEs), pain, spasticity and fatigue. Feasibility was assessed through training and support requirements and intervention fidelity. Acceptability was assessed through a satisfaction questionnaire. Impairment, activity and participation outcomes were also collected at baseline and 7 weeks to assess their suitability for use in a definitive trial.

**Randomisation:**

Computer-generated, allocation sequence concealed by opaque, sealed envelopes.

**Blinding:**

Participants and assessors were not blinded; statistician blinded for data processing and analysis.

**Results:**

192 stroke survivors were screened for eligibility, and 24 were recruited and randomised. Intervention group: n=16, mean age 66.5 years; median 9.5 days post stroke. Control group: n=8, mean age 64.6 years; median 17.5 days post stroke. Three participants withdrew before the 7-week assessment, n=21 included in the analysis (intervention group n=15; control group n=6). No significant group differences in fatigue, spasticity, pain scores or total number of AEs. The median (IQR) time to train participants was 98 (64) min over 1–3 sessions. Participants trained with the platform for a median (range) of 11 (1-58) hours, equating to 94 min extra per week. The mean satisfaction score was 34.9 out of 40.

**Conclusion:**

The Neurofenix platform is safe, feasible and well accepted as an adjunct to usual care in acute and subacute stroke rehabilitation. There was a wide range of engagement with the platform in a cohort of stroke survivors which was varied in age and level of impairment. Recruitment, training and support were manageable and completion of data was good, indicating that a future randomised controlled trial would be feasible.

**Trial registration number:**

ISRCTN11440079.

**STRENGTHS AND LIMITATIONS OF THIS STUDY**

- Participants recruited were in the acute and subacute phases post stroke and included people with higher levels of upper-limb impairment who are often under-represented in intervention trials.
- Fidelity activity data were objectively measured through the platform, thereby providing accurate data on the amount of device use.
- The trial was not designed to determine the efficacy of the virtual reality platform.
- Non-blinding of the assessors could have generated higher effect estimates.

## Introduction

 The lifetime risk of stroke for those aged 25 and over is 1 in 4,^1^ with stroke representing the third leading cause of disability worldwide.^2^ Upper-limb paresis is experienced by over two-thirds of stroke survivors.^3 4^ By 6 months, only 5–20% will experience full recovery of the upper limb^5^ and approximately 65% of all individuals post stroke will be unable to effectively use their affected upper limb for activities of daily living.^6^

The 2023 National Clinical Guideline for Stroke for the UK and Ireland recommends repetitive task practice as the principal rehabilitation approach to achieve recovery in the upper limb and that it should be commenced early after stroke and at a high intensity.^7^ The consensus in the literature, however, is that the current provision of upper-limb rehabilitation is markedly insufficient in dosage and timeliness to benefit the individual.^8^,^10^ The number of functional upper-limb movement repetitions completed during observed therapy sessions on inpatient stroke units has been reported to range from 23 to 86,^11 12^ both of which fall well below the beneficial intensities demonstrated in animal studies.^13 14^

Recovery can be enhanced by starting upper-limb rehabilitation early post stroke, with the first month being a particularly important window of opportunity^15 16^ due to a likely period of enhanced neuroplasticity.^17^ However, despite literature emerging over a decade ago that highlighted the inadequate levels of acute and subacute upper-limb rehabilitation,^18^ evidence suggests little progress has been made.^19^ In the UK, contributing factors to this limited progress are low staffing levels and organisational limitations such as limited weekend provision of therapy.^20^

**Figure 1:**
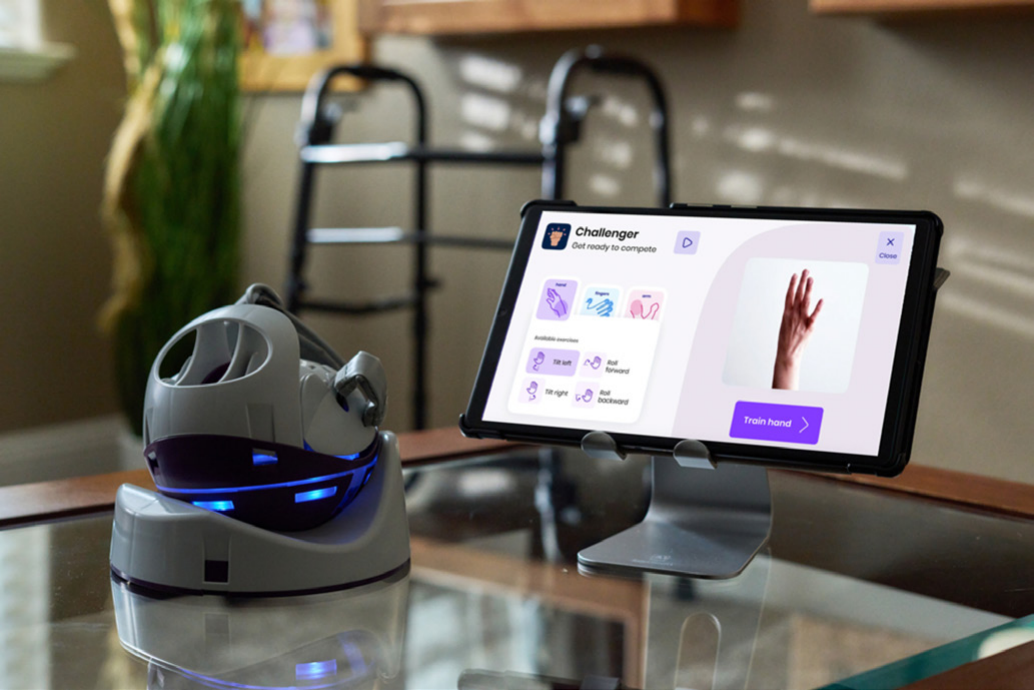
The Neurofenix platform.

With limited staff resources to deliver adequate therapy^20^ and common individual barriers to upper-limb stroke rehabilitation such as low motivation, self-efficacy and not having enough movement for meaningful practice,^21 22^ rehabilitation technology could provide a potential solution.^23 24^ Virtual reality (VR) rehabilitation platforms offering a gamified and motivating rehabilitation approach have been shown to be an effective method of increasing the intensity of upper-limb rehabilitation post stroke.^25^,^27^ The Neurofenix platform (www.neurofenix.com), referred to as ‘the VR platform’ throughout, is one such example that has been co-developed by clinical researchers, stroke survivors and bioengineers. The VR platform uses the NeuroBall, an upper-limb controlled therapy device, which translates hand and arm movements into gameplay, displayed on a tablet computer via a uniquely designed rehabilitation gaming app (figure 1). Calibration to the user’s individual level of function provides a suitable level of challenge each time the device is used, thereby providing a consistently appropriate level of challenge. The VR platform has been developed through an evidence-led and iterative process guided by the Medical Research Council Complex Interventions Framework,^28 29^ beginning with a proof-of-concept study,^30^ followed by a feasibility intervention study with community-dwelling chronic stroke survivors.^31^ The self-directed use of the VR platform at home was found to be safe, feasible and well accepted. The need for recovery trials early after stroke has been highlighted by Bernhardt and colleagues;^16^ therefore, we conducted this feasibility randomised controlled trial (RCT) in a sample of individuals in the acute and subacute stages of recovery in hospital and community settings.

### Study aims and objectives

The overall aims of this study were to determine the safety, feasibility and acceptability of the VR platform for upper-limb rehabilitation in acute and subacute stroke and assess the feasibility of conducting a definitive trial.

## Methods

The study is reported in accordance with the Consolidated Standards of Reporting Trials (CONSORT) reporting guidelines for randomised pilot and feasibility trials.^32^ Further details on trial procedures can be found in the published protocol.^33^

### Patient and public involvement

Members of two community stroke groups provided guidance on the study design and trial documentation, including the participant information sheets and consent forms. Two additional stroke survivors were on the Trial Steering Committee and were reimbursed for their time and expertise. The six principles of the UK Standards for Public Involvement were followed throughout.^34^

### Trial design

This was a feasibility RCT with a parallel process evaluation, comparing the use of the VR platform plus usual care, with usual care only.

### Participants

24 participants were recruited based on recommendations that a sample size of between 24 and 50 participants is sufficient for feasibility studies.^35 36^ Recruitment, data collection and intervention delivery took place in the Acute Stroke Unit (ASU) at Hillingdon Hospitals’ NHS Foundation Trust (London, UK) and in the homes of participant stroke survivors under the care of the Central and North West London NHS Foundation Trust Early Supported Discharge (ESD) stroke team. Recruitment involved the ASU and ESD clinical teams identifying and screening potential participants before providing eligible individuals with the participant information sheet. Interested participants were then approached by the research assistant (RA) (VS, an experienced neuro-physiotherapist) who answered any questions they had about the study and obtained written informed consent from those who wanted to take part. Inclusion criteria were (1) aged 18 years or over; (2) clinically confirmed stroke with new unilateral weakness; (3) capacity to consent; (4) mild-to-severe reduction in arm and/or hand function post stroke (Motricity Index score 9–25); (5) sufficient English to participate in the intervention and assessments and (6) able to see the graphics and visual display on the screen. Exclusion criteria were (1) unstable medical conditions; (2) unable to follow a two-stage command; (3) uncontrolled photosensitive epilepsy; (4) shoulder/arm pain exacerbated on movement; (5) fixed contracture, active disease or orthopaedic conditions affecting the hemiplegic arm; (6) current participant in an upper-limb rehabilitation trial; (7) significant cognitive impairment and inability to comprehend and follow all instructions relating to participation in the study and (8) care home residents.

### Randomisation

Following baseline assessments, participants were randomly allocated to the intervention or usual care group. A 2:1 allocation ratio was selected in accordance with the study aims to allow maximal learning about the intervention while also testing willingness to be allocated to the control group.^37^ A person independent of the delivery of the study generated a sequence using permuted blocks of randomly chosen size 3 or 6. The allocation sequence was placed in opaque, sealed envelopes. Following each baseline assessment, an envelope was drawn sequentially by the RA who informed the participant if they were in the intervention or control group.

### Intervention and comparator

Both groups received usual care for their upper-limb throughout the duration of the study, as determined by the ASU and ESD clinicians guided by the National Stroke Guidelines at the time of the study.^38 39^

In addition to usual care, the intervention group were provided with the VR platform for a 7-week intervention period. For both groups, usual care was recorded during the intervention period using a standardised form. All participants were asked not to use any other video gaming technology for their affected upper limb during the intervention period.

A detailed description of the intervention can be found in the study protocol.^33^ In summary, the VR platform is a non-immersive VR digital therapy platform consisting of the NeuroBall and tablet computer with the Neurofenix app, designed for upper-limb stroke rehabilitation. The NeuroBall is a portable sensor-enabled hand controller that tracks arm and hand movements and provides extrinsic feedback through an artificial intelligence-enabled analytics dashboard. The device promotes specific practice of unilateral or bilateral movements in the shoulder, elbow, wrist and hand through uniquely designed games displayed on a tablet computer and is supported by an instruction handbook, a QuickStart guide and short instructional videos. The software measures activity data, including games played, duration of play and the number of repetitions performed, which is automatically sent via secure transmission to bioengineers at Neurofenix. All participants allocated to the intervention group received their own personal NeuroBall and tablet computer preloaded with the app to use as a self-directed adjunct to their usual scheduled therapy session. Participants were advised to slowly increase the time spent using the device with the target of reaching or surpassing 45 min use per day as guided by the then-current National Stroke Guidelines,^38 39^ with further advice given to self-limit use if pain or fatigue were experienced.

#### Training

On receiving the VR platform, all participants, and family members when wanted, received training on how to use the device from the RA. To help guide the training, a questionnaire about previous experience with technology and gaming was completed first. The training covered technical aspects of the device, such as turning it on, accessing and navigating the app and device charging, and physical use of the device including how to don and doff the NeuroBall, maintain good posture and avoid compensatory movements. Participants who were recruited from the ASU and were transferred home with the device received a follow-up visit at home from the RA within two working days of discharge to ensure they had successfully set up the device for use at home.

To help staff support participants who were under their care, members of the ASU and ESD teams were offered optional 1-hour training sessions on using the platform and given access to recorded training material, a handbook and the study team to answer any questions that arose during the study.

### Outcomes

Assessment of outcome measures was completed by a member of the research team (a highly experienced neuro-physiotherapist) following a standardised operating procedure at baseline and 7 weeks. Assessments commenced within two working days of study enrolment, and within 2 days of the end of the intervention.

Assessors were not blind to group allocation as efficacy was not being examined in this study. A person independent of the study applied anonymous codes to all data sheets before analysis to ensure the research team and statistician were blinded to group allocation when processing and analysing the data.

Additional data collected at the baseline assessment included sociodemographic information, relevant medical history and stroke details including the National Institute of Health Stroke Scale (NIHSS) score obtained from the individual’s healthcare records.

#### Safety

Safety was monitored by assessing pain, fatigue and spasticity at baseline and 7 weeks and by collecting information on adverse events (AEs) throughout the study. A 10-point Visual Analogue Scale (VAS) was used to assess pain levels in the shoulder, elbow and wrist, with an average value for the previous 24 hours and also for the worst level of pain experienced (if any).^40^ Fatigue was assessed using the Fatigue Severity Scale (FSS-7).^41^ Higher scores indicate higher levels of pain and fatigue, respectively. The Modified Modified Ashworth Scale (MMAS) was used to assess spasticity in the shoulder adductors, internal rotators, elbow flexors, wrist flexors and finger flexors, with higher scores indicating more marked spasticity.^42 43^

#### Feasibility of intervention delivery

Feasibility of delivering the intervention was measured by recording the number and duration of training sessions the participants required. The number, duration and type (inpatient assistance, phone call or home visit) of additional points of clinical or technical support received were also recorded. Post-training confidence with the platform was assessed with a 10-point VAS (higher score equates to more confidence). The number of healthcare staff who attended training sessions on the VR platform and the number of requests for clinical and/or technical assistance related to the use of the platform with participants were also recorded.

#### Fidelity

Fidelity to the intervention was assessed using data collected automatically by the VR platform on time spent training, number of days trained on and number of upper-limb movements performed.

#### Acceptability

The eight-item Quebec User Evaluation of Satisfaction with assistive Technology (QUEST)^44^ was used to objectively measure satisfaction with the VR platform which has a total score from 8 to 40, with higher scores indicating higher satisfaction. User experience with the intervention gave further insights into acceptability, explored using semistructured interviews with a purposive sample (gender, age, amount of use, confidence with technology and level of upper-limb impairment) of 11 participants from the intervention group and 8 members of clinical staff who assisted with the delivery of the intervention for participants under their care. Interviews were conducted by the RA following a topic guide developed from the seven-component constructs of the Theoretical Framework of Acceptability.^45^ Qualitative results will be reported in a separate paper.

#### Feasibility of a definitive trial

Response rate, retention, outcome measure completion and reasons for missing data were recorded to assess the feasibility of a definitive trial.

The following outcome measures were used to assess their suitability for use in a definitive trial. Arm function was assessed using the Action Research Arm Test (ARAT); scores range from 0 to 57, with higher scores indicating better function.^46 47^ Self-reported arm function was assessed using the 14-item Motor Activity Log (MAL-14), with higher scores indicating greater functional use of the upper limb.^48^ Upper-limb impairment was assessed using the Fugl-Meyer Assessment—Upper Extremity (FMA-UL); scores range from 0 to 60, with higher scores indicating less impairment.^49 50^ Passive range of movement (PROM) of the shoulder (flexion, abduction, external rotation), elbow (flexion and extension) and wrist (extension) was assessed using handheld goniometry.^51^

The simplified modified Ranking Scale questionnaire (smRSq) was used to measure global function, with a higher score representing greater disability (range 1–5).^52 53^ The 10-item Subjective Index of Physical and Social Outcome (SIPSO) was used to measure participation, with higher scores indicating increased ability to reintegrate to a ‘normal’ lifestyle.^54^ Self-efficacy was measured using the Self-Efficacy for Home Exercise Programs Scale (SEHEPS) (range 0–72), with higher scores indicating higher self-efficacy.^55^ Anxiety and depression were measured using the Generalised Anxiety Disorder 2-item (GAD-2)^56^ and the Patient Health Questionnaire-2 (PHQ-2),^57^ with higher scores indicating more severe anxiety and depression, respectively. Quality of life was assessed using the EuroQol 5 Dimensions 5 levels questionnaire (EQ-5D-5L).^58^ A utility value (ie, a score) was calculated as recommended using the cross-walk function which maps the EQ- 5D-3L and newer EQ- 5D-5L questionnaires,^59^ with higher values indicating better quality of life. A modified version of the Client Service Receipt Inventory was used to assess health service use.^60^

### Data analysis

All data were analysed using Jamovi V.2.3.28. Participant characteristics were described using mean, SD, median, IQR, frequency and percentage as appropriate. Similarly, safety-related outcomes and outcomes related to the feasibility of the intervention, fidelity and acceptability were all reported using descriptive statistics. For safety-related outcomes, between-group differences were examined using χ^2^ for categorical outcomes (pain) and analysis of covariance (ANCOVA) for continuous outcomes (fatigue and spasticity, controlling for baseline score of the respective outcome). Candidate primary and secondary outcome measures were examined by comparing outcomes (ARAT, FMA-UL, PROM, smRSQ, SIPSO, MAL-14, EQ-5D-5L, GAD-2, PHQ-2 and SEHEPS) assessed post intervention between groups using ANCOVA adjusted for baseline score.

## Results

**Figure 2:**
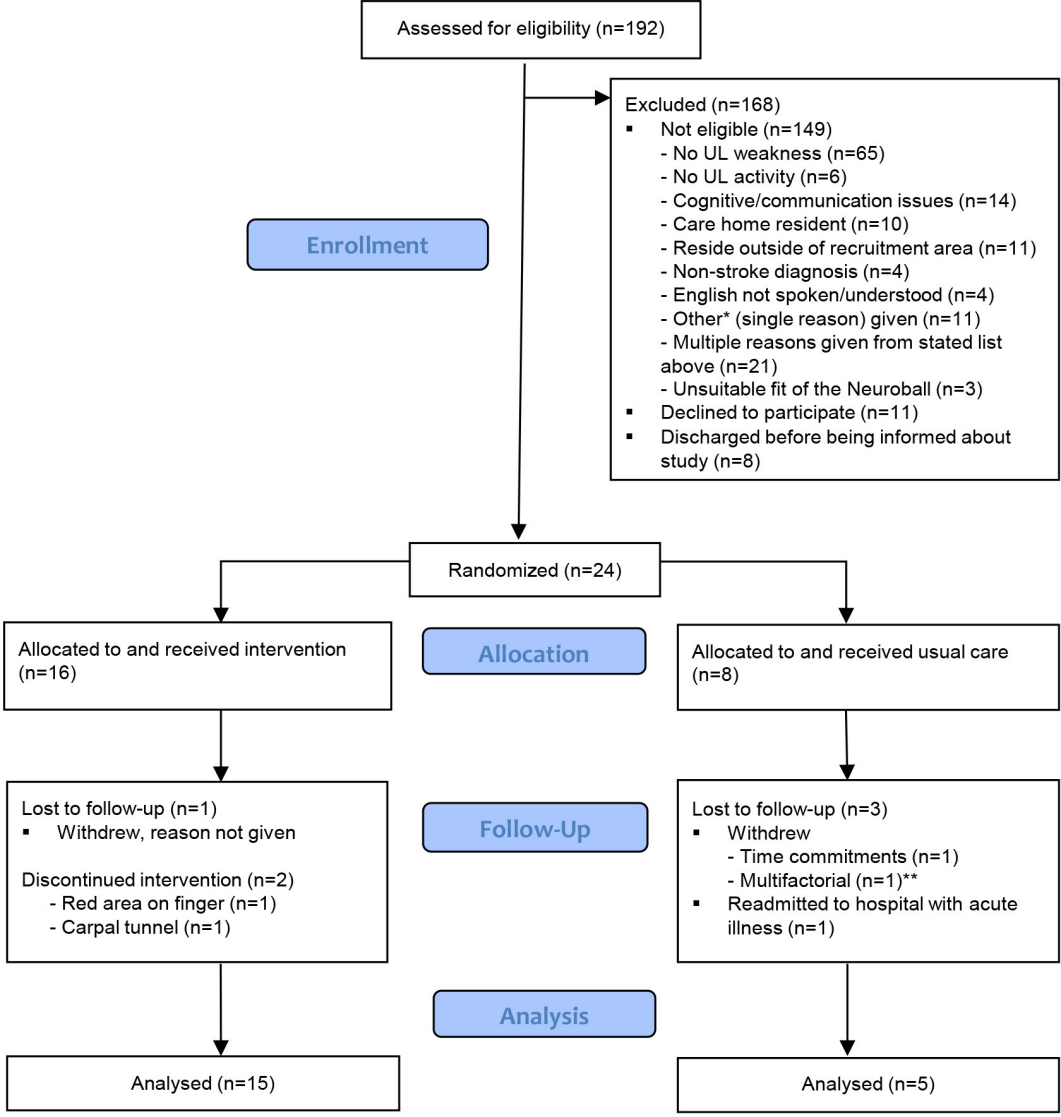
Consolidated Standards of Reporting Trials (CONSORT) flow of participants through the trial. UL, upper limb. *Other: bedbound, pain, increased tone, medically unwell, significant mental health disorder, premorbid cognition, rousability, existing participant, refusing Early Supported Discharge (ESD) visits, complex social history. **Multifactorial: dissatisfaction with allocation to the control group alongside high levels of poststroke anxiety and depression.

24 participants were recruited in the study between April 2021 and January 2022 out of 192 who were assessed for eligibility (figure 2). The trial ended when data collection was complete for all recruited participants (February 2022). In the intervention group (n=16), participants (13 women) had a mean age (SD) of 66.5 (15) years (minimum-maximum: 35–89) and were a median of 9.5 days post stroke (minimum-maximum: 1–42 days). In the control group (n=8), participants (four women) had a mean age (SD) of 64.6 (13.6) years (minimum-maximum: 41–79) and were a median of 17.5 days post stroke (minimum-maximum: 4–23 days). Baseline scores for the smRSQ, FMA-UE and ARAT were broadly similar between both groups (table 1).

**Table 1:** Baseline characteristics of all participants in the intervention (n=16) and control (n=8) groups

	Intervention group (n=16)				Control group (n=8)			
	N (%)	Mean (SD)	Median (IQR)	Range	N (%)	Mean (SD)	Median (IQR)	Range
Age, year	16	66.5 (15.0)	68.0 (20.3)	35–89	8	64.6 (13.6)	68.0 (17.8)	41–79
Women	13 (81%)				4 (50%)			
Ethnicity								
White	14 (87.5%)				6 (75%)			
Asian	2 (12.5%)				1 (12.5%)			
Black	0 (0%)				1 (12.5%)			
Time since stroke, days	16	17 (13.9)	9.5 (16.5)	1–42	8	16 (6.7)	17.5 (9.5)	4–23
Previous stroke (yes)	5 (31.2%)				1 (12.5%)			
Stroke type (self-report)								
Haemorrhagic	5 (31.25%)				2 (25%)			
Ischaemic	10 (62.5%)				5 (62.5%)			
Not known	1 (6.25%)				1 (12.5%)			
smRSQ	16	3.5 (1.0)	3.0 (1.0)	2–5	8	3.5 (1.1)	3.0 (1.3)	2–5
FMA-UE (0–66)	16	36.1 (18.1)	43.0 (29.3)	3–55	8	41.5 (18.6)	51.0 (14.5)	4–56
ARAT (0–57)	16	29.5 (21.0)	36.0 (42.5)	0–56	8	36.1 (22.7)	47.5 (27.8)	0–55
MMAS – worse score (0–5)	16	0.8 (0.9)	0.5 (1.3)	0–2	8	0.4 (0.5)	0 (1.0)	0–1
NIHSS	16	6.6 (2.7)	6.0 (3.0)	3–13	8	5.6 (3.4)	5.0 (2.5)	2–13
Used tablet/computer/ console/smartphone					N/A			
Never	9 (56%)							
Occasionally	6 (38%)							
Often	1 (6%)							
Always	0 (0%)							
Confidence pre-training with the VR platform (0–10)	16	4.5 (2.2)	4.5 (2.3)	0–10	N/A			
Motivation pre-training with the VR platform (0–10)	16	7.1 (1.7)	7.0 (1.3)	3–10	N/A			

ARAT, Action Research Arm Test; FMA-UE, Fugl-Meyer Assessment Upper Extremity; MMAS, Modified Modified Ashworth Scale; NIHSS, National Institute of Health Stroke ScalesmRSQ, simplified modified Rankin Scale Questionnaire

### Safety

There were no differences in score on the FSS-7 (fatigue) and MMAS (spasticity), respectively, between groups at post intervention (table 2). The site of pain most reported by participants was the shoulder, with both groups seeing an increase in reported shoulder pain from baseline to postintervention assessment (table 3). χ^2^ tests revealed no significant between-group differences in the shoulder, elbow and wrist pain prevalence at postintervention assessment.

**Table 2:** Scores on fatigue and spasticity at baseline and post intervention for both groups

	Intervention group (n=16)		Control group (n=8)		Group difference post intervention	
	Baseline	Post intervention*	Baseline	Post intervention†		
	Mean (SD)(range)	Mean (SD)(range)	Mean (SD)(range)	Mean (SD)(range)	Adj. mean difference (95% CI)	P value
Fatigue—FSS-7 (7–49)	28.5 (11.3) (7–44)	24.1 (11.1) (8–49)	36.6 (11.6) (16–47)	32.4 (12.4) (14–44)	4.9 (−6.3, 16.2)	0.401
Spasticity—MMAS (0–5)	0.8 (0.9) (0–2)	1.1 (0.6) (0–3)	0.4 (0.5) (0–1)	0.4 (0.6) (0–1)	0.1 (−0.7, 1.0)	0.760

* 1One missing case.

† 3Three missing cases.

FSS-7Fatigue Severity ScaleMMASModified Modified Ashworth Scale

**Table 3:** Pain occurrence (%) and average pain scores at baseline and post intervention for both groups

	Intervention group (n=16)		Control group (n=8)		Group difference post intervention (χ^2^)	
	Baseline	Post intervention*	Baseline	Post intervention†		
	N (%)/median (IQR)	N (%)/median (IQR)	N (%)/median (IQR)	N (%)/median (IQR)	Χ^2^ (df)	P value
Shoulder pain (yes/no)	3 (19%)	6 (40%)	1 (13%)	2 (40%)	Χ^2^ (1) = 0.07	0.787
Shoulder pain VAS-max	7 (2.5)	7 (3.5)	3	7.5 (0.5)		
Shoulder pain VAS-av	3 (3.5)	4 (4.3)	3	3.5 (3.5)		
Elbow pain (yes/no)	0 (0%)	1 (6%)	0 (0%)	0 (0%)	Χ^2^ (1) = 0.35	0.554
Elbow pain VAS-max	N/A	9	N/A	N/A		
Elbow pain VAS-av	N/A	8	N/A	N/A		
Wrist pain (yes/no)	1 (6%)	4 (27%)	0 (0%)	0 (0%)	Χ^2^ (1) = 1.67	0.197
Wrist pain VAS-max	7	7 (2.3)	N/A	N/A		
Wrist pain VAS-av	6	1.5 (4.3)	N/A	N/A		
Pain—any other location (yes/no)‡	1 (6%)§	3 (20%)¶	0 (0%)	1 (20%)**	Χ^2^ (1) = 0.00	1.000
Pain—any other location; VAS-max	5	8 (3.5)	N/A	2		
Pain—any other location; VAS-av	4	3 (3.5)	N/A	N/A††		

* 1One missing case.

† 3Three missing cases.

‡ “’Pain— - any other location’ concerns pain reported for either the upper- arm, lower- arm, hand, fingers, neck or trunk. Shown here is the number of participants thatwho experienced pain at any (or multiple) of these locations. Subdivision was as follows.

§ Upper arm and fingers (n=1).

¶ Unspecified pain in lower back (n=1), Tthumb (n=1), Hhand (n=1).

** Neck (n=1).

†† Participant found it difficult to rate average pain.

VAS-av, Visual Analogue Scale (average pain over last 24 hours)VAS-max, Visual Analogue Scale (worst level of pain)

In total, there were eight AEs, three of which were serious (SAEs), reported by a total number of seven (29%) participants (online supplemental table 1). Five AEs were in the intervention group, one (red area on the third finger) was considered to definitely be related to the intervention, one (carpal tunnel type symptoms) was considered probably related, while the remaining three (shoulder pain, thumb pain and migraine) were possibly related, these were all mild and potentially expected AEs. There was one SAE in the intervention group involving nausea, vomiting and visual disturbance. Following discussion with the trial stroke consultant (BA), this SAE was judged to be possibly, but unlikely related to the intervention due to the unrelated timing of symptom onset with device use, as well as the symptoms being linked to an ongoing viral infection. The two SAEs in the control group concerned one participant who required admission to hospital for reasons unrelated to the study. A negative binomial model revealed no significant difference between groups in the total number of AEs (β=0.405, 95% CI = [−1.41, 2.22], p=0.661).

### Feasibility of the intervention

All participants in the intervention group (n=16) completed training on how to use the platform (online supplemental table 2). Eight participants (50%) needed one training session, five (31%) needed two sessions and three (19%) needed a third session. The median time for training, over 1–3 sessions, was 98 min per participant (IQR=64; range: 45–200 min). On training completion, participant confidence with the device was a median (SD) of 7.2 (2.1) out of 10 (range: 2–10). 14 members of staff from the ASU and ESD teams attended training on the intervention prior to the study commencing.

Support provided during the intervention period was grouped as clinical support (eg, regarding management of pain, tone), support for device use (eg, reattaching straps, logging in) or technical issue resolution (eg, app software glitches). Four (25%) participants required no assistance. Of the 12 (75%) participants requiring assistance, 2 required all three types of support, 4 required two types and 6 needed only a single type of support. Detailed information on the support provided is reported in online supplemental table 3.

### Fidelity

Participants trained with the VR platform for a median (minimum-maximum) of 11 (1–58) hours over the 7-week intervention period, equating to a median of 94 min per week. Participants trained for a mean (SD) of 22.4 (14.4) days over the 7 weeks (range: 3–48 days) equating to a mean of 3.2 (1.9) days per week. Two (12.5%) participants completed an average of more than 225 min per week which would have met the then recommended amount of 45 min per day, 5 days a week.^39^ In total, participants performed a median of 10 276 (range: 1586–71 511) upper-limb movements over the 7-week period, equating to 210 repetitions each day.

### Acceptability and factors impacting adoption

For participants in the intervention group, the mean (SD) score on the QUEST was 34.9 (4.3) out of 40 (range: 26–40), indicating a high level of user satisfaction with the VR platform.

### Preliminary effects

The mean differences in outcomes for each group between baseline and post intervention are reported in table 4, including the between-group comparison scores at the postintervention assessment. Both groups demonstrated improvements across the range of measures; however, there were no statistically significant differences in any outcomes between groups at 7 weeks.

**Table 4:** Outcomes at baseline and post intervention for both groups

Outcome	Intervention group (n=16)			Control group (n=8)			Group difference post intervention	
	Baseline	Post intervention*	Mean change	Baseline	Post intervention†	Mean change		
	Mean (SD)	Mean (SD)	Mean difference (95% CI)	Mean (SD)	Mean (SD)	Mean difference (95% CI)	Adj. mean difference(95% CI)	P value
ARAT (0–57)	29.5 (21.1)	38.2 (20.1)	8.9 (2.4, 15.4)	36.1 (22.7)	43.8 (24.2)	5.6 (−2.7, 13.9)	−1.8 (−12.6, 9.0)	0.748
FMA-UE (0–66)	36.1 (18.1)	44.4 (16.7)	8.3 (4.3, 12.3)	41.5 (18.6)	49.8 (17.4)	8.6 (2.0, 15.2)	1.3 (−4.9, 7.4)	0.697
smRSQ (1–5)	3.5 (1.0)	2.8 (0.9)	−0.7 (1.2, 0.2)	3.5 (1.1)	2.6 (1.5)	−0.6 (−1.7, 0.5)	0.0 (−0.8, 0.9)	0.941
SIPSO (total score; 0–40)	14.0 (6.9)	21.2 (8.2)	7.3 (4.5, 10.1)	13.0 (5.0)	21.8 (11.4)	8.8 (−2.2, 15.5)	1.6 (−3.8, 7.0)	0.570
MAL (0–5)	1.2 (1.3)	2.2 (1.5)	1.0 (0.5, 1.5)	1.6 (1.2)	2.8 (2.1)	1.1 (0.1, 2.2)	0.1 (−0.8, 1.0)	0.843
EQ-5D-5L; utility score	0.22 (0.36)	0.47 (0.37)	0.28 (0.15, 0.40)	0.20 (0.24)	0.27 (0.40)	0.10 (−0.07, 0.27)	−0.18 (−0.40, 0.03)	0.120
GAD-2 (0–6)	2.4 (2.4)	1.4 (2.2)	−0.9 (−1.8, 0.1)	2.3 (2.3)	2.0 (2.5)	−0.3 (−3.1, 2.6)	0.6 (−1.1, 2.4)	0.490
PHQ-2 (0–6)	2.2 (2.2)	1.1 (2.1)	−1.1 (−1.9, 0.4)	1.9 (2.1)	0.9 (1.3)	−1.0 (−3.0, 1.0)	0.0 (−1.3, 1.2)	0.980
SEHEPS	32.3 (17.7)	28.3 (14.0)	−5.9 (−2.1, 13.9)	30.1 (21.1)	39.8 (11.8)	6.6 (−28.2, 15.0)	12.0 (−0.1, 24.1)	0.070
Shoulder flexion	145.9 (37.0)	144.0 (43.2)	−1.3 (−28.4, 25.7)	145.0 (32.4)	159.0 (34.7)	16.0 (−40.0, 71.9)	15.7 (−26.0, 57.4)	0.471
Shoulder external rotation	52.5 (18.0)	54.3 (26.9)	−0.7 (−15.8, 14.5)	57.1 (18.7)	63.0 (17.9)	8.6 (−16.5, 33.7)	8.3 (−16.6, 33.2)	0.519
Shoulder abduction	121.6 (42.2)	135.7 (45.7)	12.0 (−12.9, 36.9)	127.5 (45.6)	140.0 (40.6)	20.0 (−30.4, 70.4)	6.2 (−33.6, 46.0)	0.764
Elbow flexion	136.3 (11.9)	133.9 (13.8)	−1.4 (−6.0, 3.2)	143.8 (6.9)	143.0 (8.4)	−1.0 (−10.2, 8.2)	3.0 (−5.5, 11.5)	0.498
Elbow extension	−0.9 (6.9)	−1.33 (5.2)	−0.7 (−5.1, 3.8)	0.0 (2.7)	2.0 (4.5)	2.0 (−7.4, 11.4)	3.3 (−1.9, 8.5)	0.232
Wrist extension	65.3 (18.8)	63.0 (17.4)	−2.3 (−10.0, 5.4)	74.4 (14.3)	76.0 (26.8)	2.0 (−23.1, 27.1)	6.8 (−8.8, 22.3)	0.405

All post-test group comparisons were corrected/adjusted for baseline score.

Range of motion test results concerned passive range of movement, in degrees.

* 1One missing case, except for GAD-2 and PHQ.

† 3Three missing cases, except for GAD-2 and PHQ for which data was available for the entire sample.

ARAT, Action Research Arm Test; EQ-5D-5L, EuroQol 5 Dimensions 5 Levels; FMA-UE, Fugl-Meyer Assessment—Upper Extremity; GAD-2, Generalised Anxiety Disorder (two-item version); MAL, Motor Activity Log; PHQ-2, Patient Health Questionnaire (two-item version); SEHEPS, Self-Efficacy for Home Exercise Programme Scale; SIPSO, Subjective Index of Physical and Social Outcome; smRSQ, Simplified Modified Rankin Scale Questionnaire

### Feasibility of conducting a definitive trial

The recruitment rate was 12.5%. As outlined in figure 2, 192 people were assessed for study eligibility, of which 149 were not eligible, 8 were discharged before being informed about the study and 11 declined to participate. Three (19%) participants in the intervention group did not complete the full 7-week intervention, with two of these being due to AEs and the other because the participant withdrew from the study (declined to give reason). Two participants in the control group also withdrew from the study (multifactorial and time commitment). All participants completed all outcome measures at baseline. The three participants who withdrew from the study did not complete the follow-up assessments. One additional participant in the control group did not complete the follow-up assessments due to being medically unwell, resulting in an 83% retention at the 7-week assessment; additionally, breakdown scores for the NIHSS were missing for six participants due to the information not being recorded in their medical notes by the assessing clinician. Limited information was available from documentation on the specific intervention types and dosage of ‘usual care’ for both groups, with COVID-19 restrictions further impacting the ability to visit the Stroke Unit to gather data on this.

## Discussion

The results of this feasibility RCT demonstrate the VR platform to be a safe, feasible and acceptable intervention for rehabilitation of the upper limb for individuals with acute and subacute stroke with mild-to-severe upper-limb impairment.

On average, participants in the intervention group trained with the VR platform, as an adjunct to usual care, more than 3 days per week, for 94 min per week, achieving 41% of the then-current recommendation of 45 min per day, 5 days per week through device use alone.

Two participants completed over 225 min per week, thereby achieving this recommendation by using the device. The 94 min averaged per week is lower than the 149 weekly minutes averaged by participants in the RHOMBUS I trial.^31^ This may reflect the fact that participants in the RHOMBUS I trial were chronic stroke survivors receiving no therapy input during the trial, whereas participants in this study were acute or subacute stroke survivors using the platform in addition to their usual care from the inpatient or ESD therapy teams. While 11 hours of upper-limb rehabilitation and 10 276 repetitions of movement over the 7-week intervention period falls short of the recommended 15–30 hours of VR rehabilitation,^61^ it does reflect a marked increase on the low levels of upper-limb rehabilitation currently reported in subacute stroke rehabilitation.^9 62^ Given the positive dose-response relationship between practice and recovery, any increase in practice time could therefore be beneficial.^63^

Collection and reporting of outcome data were very good, with missing outcome data only being due to withdrawal, not difficulty undertaking the assessments, indicating that it would be acceptable and feasible to use these measures in a future RCT. The four core measures for stroke trials were used, following the recommendations of the Stroke Recovery and Rehabilitation Roundtable.^64^ This included the ARAT, which saw a mean change of 8.9 points in the intervention group and 5.6 points in the control group. The ARAT has a minimally clinically important difference of six points for those early after stroke,^65^ suggesting that it could be a suitable and sensitive measure to be used as the primary outcome measure in a future RCT.

The variable levels of adherence seen in this study are consistent with previous literature on upper-limb rehabilitation technologies^66^,^68^ and highlight the need for further work which investigates motivation and adherence given the potential benefits these technologies are known to offer. Yoshida *et al*^69^ explored factors impacting rehabilitation motivation in subacute stroke and identified several core categories, including patients’ goals and experience of success and failure. Within the context of these findings, it is understandable how the VR platform has the capacity to improve adherence as the gaming components allow for goals to easily be set either with in-game scores or difficulty levels, or time-orientated goals based on how much time was spent using the device each day, both of which have previously been identified as factors motivating persistence with the platform.^70^ Experience of success and failure can impact motivation in subacute stroke positively or negatively, respectively,^69^ which could be a contributing factor to the variability of adherence levels seen with VR rehabilitation devices; however, the platform used in this study attempted to minimise the experience of failure by calibrating to the individual user’s level of function on each use of the device, adjusting the level of challenge appropriately to maximise the likelihood of success within the games.

Our findings on safety are consistent with the findings of the 2017 Cochrane Review^23^ that VR is a safe approach for stroke rehabilitation, with AEs considered to be possibly or probably related to the platform being predominantly mild. Frequency of shoulder pain reporting increased in both the intervention group and the control group at the follow-up assessment which is not unexpected given the general risk of poststroke shoulder pain increases with time since stroke onset.^71^ Therefore, while the platform did not reduce the risk of shoulder pain in subacute stroke in the way that it did in chronic stroke,^31^ it does not appear to have increased the risk.

Recruitment targets were achieved within the specified timeframe of 10 months, with 12.5% of those assessed for eligibility being enrolled in the trial. Comparatively this is a strong recruitment rate compared with many randomised subacute stroke rehabilitation trials which tend to achieve single-digit recruitment rates.^72 73^ Achieving the target of 10-month recruitment timeframe for the 24 participants also compares favourably with a similar UK-based upper-limb VR randomised controlled feasibility trial which took 15 months to recruit 27 participants.^74^ Of the 24 participants who completed baseline measures, 20 (83%) completed the follow-up measures, giving an acceptable attrition rate of less than 20%,^75^ which is directly comparable to figures from Adie *et al*^76^ but improved on the 67% retention rate reported by Standen *et al*.^74^ Both of these randomised trials were conducted in the home environment, unlike this study which took place both in hospital and at home for those with acute and subacute stroke, which is novel within the research field, meaning a direct comparison to a similar study cannot be made. Acceptability of allocation to the control group also appears good, with two (25%) participants withdrawing from the control group and only one of those citing allocation to the control group as being a contributing factor.

Half of the participants required only one training session to feel confident using the device, with the remaining half needing either two or three training sessions, which differs from the RHOMBUS I trial^31^ in which most participants only required one training session. The median time taken to complete the training, regardless of the number of sessions it took, in both this study and the RHOMBUS I trial was 98 min, suggesting that those in the subacute stage post stroke do not require increased training time, but may require multiple shorter training sessions than those in the chronic stage.

A strength of this study is the broad range of participants, including those with mild-to-severe levels of impairment with acute and subacute stroke who are often under-represented in stroke rehabilitation research.^16 77^ As with many studies that have looked at the use of gaming technologies for rehabilitation, at 66.5 years of age the average age of participants in the intervention group is lower than the average age of stroke reported as being 71.4 years of age; however, there was a wide age range from 35 to 89 years of age, with older participants not excluded from taking part as has been reported to be the case in the majority of RCTs.^77^ Participants in the intervention group also had very little prior experience with technology, with only one individual reportedly using a tablet, computer, console or smartphone often prior to taking part, and over 50% of participants reporting that they never used these devices.

### Study limitations

Methodological limitations of this study include non-blinding of the assessors which could have generated higher effect estimates^78^ and also the lack of data which were able to be collected on the dose and interventions delivered as usual care in the control group.

## Conclusion

The VR platform was overall demonstrated to be safe, feasible and well accepted as an adjunct to usual care in acute and subacute stroke rehabilitation. There was a wide range of engagement with the VR platform; however, through its use, rehabilitation intensity for the UL was increased in a cohort of stroke survivors which was varied in age and level of impairment. Recruitment timeframes were met, training and support requirements were manageable and comparable to previous studies and data loss and attrition were within acceptable levels, indicating that a future RCT would be feasible.

## supplementary material

10.1136/bmjopen-2024-089672 online supplemental file 1

## Data Availability

Data are available upon reasonable request.
