# Supplementary material for: Rehabilitation using virtual gaming for Hospital and hOMe-Based training for the Upper limb in acute and subacute Stroke (RHOMBUS II): results of a feasibility randomised controlled trial
Source: BMJ Open. 2025 Jan 28;15(1):e089672. doi: 10.1136/bmjopen-2024-089672 (PMC11781105; doi:10.1136/bmjopen-2024-089672)
Supplement: online supplemental file 1 [file bmjopen-15-1-s001.docx]

**RHOMBUS II Supplemental File**

| **Supplemental Table 1.** (Serious) Adverse Events recorded during the study. | | | | | | | | | | | | | | | | |
| --- | --- | --- | --- | --- | --- | --- | --- | --- | --- | --- | --- | --- | --- | --- | --- | --- |
|  |  | | **Intervention group (n=16)** | | | | |  |  | | **Control group (n=8)** | | | | |  |
|  |  |  | | **Attribution** | | | |  | |  | | **Attribution** | | | |  |
| **Event** | **No of events** | **No of people reporting** | | **Definitely related** | **Probably related** | **Possibly related** | **Unrelated** | **No of events** | | **No of people reporting** | | **Definitely related** | **Probably related** | **Possibly related** | **Unrelated** |  |
| SAE | 1 | 1 | | 0 | 0 | 1 | 0 | 2 | | 1 | | 0 | 0 | 0 | 2 |  |
| AE | 5 | 5 | | 1 | 1 | 3 | 0 | 0 | | 0 | | 0 | 0 | 0 | 0 |  |
| Total | 6 | 6 | | 1 | 1 | 4 | 0 | 2 | | 1 | | 0 | 0 | 0 | 2 |  |

| **Supplemental Table 2. Training sessions required per participants and duration of training** | | | | |
| --- | --- | --- | --- | --- |
|  | **No of participants, n (%)** | **Duration, total no of minutes across training sessions** | | |
|  |  | *Mean (SD)* | *Median (IQR)* | *Min – Max* |
| **Training Sessions** | | | | |
| *One session* | 8 (50%) | 59 (15) | 58 (14) | 45-90 |
| *Two sessions* | 5 (31%) | 145 (42) | 120 (60) | 105-200 |
| *Three sessions* | 3 (19%) | 145 (35) | 130 (33) | 120-185 |
|  |  |  |  |  |
| **Supplemental Table 3. Total number and duration of remote (phone calls, texts, emails) and face-to-face (hospital or home visits) contact moments for clinical, device use, or technology problems related questions.** | | | | |
|  | **Total no of calls/visits (n)** | **Duration, minutes per call/visit** | | |
|  |  | *Mean (SD)* | *Median (IQR)* | *Min – Max* |
| **Clinical** | | | | |
| *Phone calls* | 4 | 7 (2) | 6.5 (2) | 5-9 |
| *Texts or Emails* | 5 | 7 (3) | 5 (2.5) | 5-10 |
| *Home visits* | 5 | 63 (11) | 60 (11) | 53-75 |
| *Hospital visits^a^* | 9 | 58 (20) | 64 (17) | 30-75 |
| *HCP F2F contact* | 1 | 20 (0) | 20 (0) | 20-20 |
| **Device use** |  |  |  |  |
| *Phone calls* | 5 | 10 (5) | 10 (5) | 5-15 |
| *Texts or Emails* | 3 | 4.3 (0) | 4.3 (0) | 4.3-4.3 |
| *Home visits* | 1 | 60 (0) | 60 (0) | 60-60 |
| *Hospital visits* | 0 | n/a | n/a | n/a |
| *HCP F2F contact* | 4 | 14 (5) | 12.5 (5) | 10-20 |
| **Technical issues** |  |  |  |  |
| *Phone calls* | 6 | 12 (3) | 10 (2.5) | 10-15 |
| *Texts or Emails* | 5 | 9 (0) | 9 (0) | 9-9 |
| *Home visits* | 0 | n/a | n/a | n/a |
| *Hospital visits* | 6 | 102 (93) | 74 (108) | 30-230 |
| *HCP F2F contact* | 6 | 21 (21) | 10 (19) | 6.6-45 |
| ^a^missing data for one participant HCP = Healthcare practitioner; F2F = face-to-face | | | | |
