# Supplementary material for: Rehabilitation using virtual gaming for Hospital and hOMe-Based training for the Upper limb in acute and subacute Stroke (RHOMBUS II): results of a feasibility randomised controlled trial
Source: BMJ Open. 2025 Jan 28;15(1):e089672. doi: 10.1136/bmjopen-2024-089672 (PMC11781105; doi:10.1136/bmjopen-2024-089672)
Supplement: Supplementary file 2 [file bmjopen-2024-089672.reviewer_comments.pdf]

PEER REVIEW HISTORY

BMJ Open publishes all reviews undertaken for accepted manuscripts. Reviewers are asked to complete a checklist review form and are provided with free text boxes to elaborate on their assessment. These free text comments are reproduced below.

ARTICLE DETAILS

Title (Provisional)

Rehabilitation using virtual gaming for Hospital and hOMe-Based training for the Upper limb in acute and subacute Stroke (RHOMBUS II): results of a feasibility randomised controlled trial.

Authors

Butcher, Tom; Warland, Alyson; Stewart, Victoria; Aweid, Basaam; Samiyappan, Arul; Kal, Elmar; Ryan , Jennifer; Athanasiou, Dimitrios A.; Baker, Karen; Singla-Buxarrais, Guillem; Anokye, Nana; Pound, Carole; Gowing, Francesca; Norris, Meriel; Kilbride, Cherry

---

VERSION 1 - REVIEW

**Reviewer**	**1**
**Name**	**Or, Calvin Kalun**
**Affiliation**	**The University of Hong Kong, Industrial and Manufacturing Systems Engr.**
**Date**	**16-Sep-2024**
**COI**	**None**

The paper is about a feasibility RCT to examine the use of VR for upper-limb rehab. My comments are as follows.

For randomization, why was a 2:1 ratio used? Why not just 1:1?

The authors mentioned that “Both groups received usual care for their upper-limb …” Please provide details regarding when the intervention and control subjects received usual care and when the usual care ended, as well as when the subjects received the technology and when it ended. Was usual care provided to all subjects in both groups at baseline (I guess so), or something else? Did all usual care stop at week 7, or something else? What about the technology intervention? Was it provided to intervention subjects at baseline, and did subjects stop receiving it at week 7? Or was the technology provided at week 7 (i.e., 7 weeks after baseline), with subjects required to use it for 7 weeks from that point? Please revise the manuscript for it.

There is a similar study, please mention it, at least for similarities and differences:

https://journals.sagepub.com/doi/full/10.1177/20552076231203599

For "… withdrew from the study (multifactorial and time commitment) …", what were the specific factors for "multifactorial". Please provide the specific details.

The authors stated that "There was one SAE in the intervention group involving nausea, vomiting and visual disturbance which was possibly, but unlikely related to the intervention." How do the authors know that the SAE was unlikely related to the intervention?

The authors can consider providing a discussion on the acceptance of VR technology-based rehabilitation exercise protocol. These papers can be mentioned:
https://www.sciencedirect.com/science/article/abs/pii/S0169814122001019

https://www.sciencedirect.com/science/article/pii/S2451958821000890

**Reviewer**	**2**
**Name**	**Lansberg, Maarten**
**Affiliation**	**THE STANFORD UNIVERSITY MEDICAL CENTER, NEUROLOGY**
**Date**	**18-Oct-2024**
**COI**	**None**

This is an exceptionally well written paper on a feasibility study of a VR intervention for stroke rehabilitation. The investigators succeeded in recruiting study particpants in a very reasonable time (10 months) and they describe the safety and feasibility of using a VR intervention for stroke rehabilitation. I only have a few minor suggestions for further improvement of the manuscript:

1. The authors could include an illustration of the VR system (ball and tablet) to provide the reader with a visual of the intervention

2. The team found that the vast majority of particpants do not reach the target goal of 45 minutes of use 5 days per week. Since the number and duration of sessions was listed as the main feasibility outcome, the conclusions in abstract and in the main text could highlight this finding which is consistent with other interventions aimed at increasing rehabilitation dose.

VERSION 1 - AUTHOR RESPONSE

All line numbers referenced correlate with the Main Document (clean version). **Black bold text** indicates reviewer comments, text in red indicates our responses/actions taken.

Reviewer: 1
Dr. Calvin Kalun Or, The University of Hong Kong
Comments to the Author:
The paper is about a feasibility RCT to examine the use of VR for upper-limb rehab. My

comments are as follows.

Thank you Dr Kalun for your review and comments, please see our responses below.

1) For randomization, why was a 2:1 ratio used? Why not just 1:1?

We have added a sentence and supporting reference in lines 223-225 which clarifies this "A 2:1 allocation ratio was selected in accordance with the study aims, to allow maximal learning about the intervention whilst also testing willingness to be allocated to the control group [37]."

2) The authors mentioned that "Both groups received usual care for their upper-limb ..." Please provide details regarding when the intervention and control subjects received usual care and when the usual care ended, as well as when the subjects received the technology and when it ended. Was usual care provided to all subjects in both groups at baseline (I guess so), or something else? Did all usual care stop at week 7, or something else? What about the technology intervention? Was it provided to intervention subjects at baseline, and did subjects stop receiving it at week 7? Or was the technology provided at week 7 (i.e., 7 weeks after baseline), with subjects required to use it for 7 weeks from that point? Please revise the manuscript for it.

Please see lines 231-256 under the section titled "**intervention and comparator**" where this information is provided. We have also amended the sentence quoted within the comment in line 232-234 to read "Both groups received usual care for their upper-limb throughout the duration of the study, as determined by..."

3) There is a similar study, please mention it, at least for similarities and differences:
https://journals.sagepub.com/doi/full/10.1177/20552076231203599

Thank you for providing the suggested study, however as it involved the use of immersive virtual reality via a head mounted display which often poses different challenges regarding acceptability and feasibility to non-immersive virtual reality, which was used in our study, we have made the decision not to reference the suggested study on this occasion.

4) For "... withdrew from the study (multifactorial and time commitment) ...", what were the specific factors for "multifactorial". Please provide the specific details.

Additional details have been provided by adding a double asterisk next to "multifactorial" in the CONSORT table (table 2), with additional information provided the figure legend which can be seen in line 927 "**Multifactorial: Dissatisfaction with allocation to the control group alongside high levels of post-stroke anxiety and depression."

5) The authors stated that "There was one SAE in the intervention group involving nausea, vomiting and visual disturbance which was possibly, but unlikely related to the intervention." How do the authors know that the SAE was unlikely related to the intervention?

We have added additional detail to this statement in lines 408-411, now stating "Following discussion with the trial stroke consultant (BA) this SAE was judged to be possibly, but unlikely related to the intervention due to the unrelated timing of symptom onset with device use, as well as the symptoms being linked to an ongoing viral infection."

6) The authors can consider providing a discussion on the acceptance of VR technology-based rehabilitation exercise protocol. These papers can be mentioned:
https://www.sciencedirect.com/science/article/abs/pii/S0169814122001019
https://www.sciencedirect.com/science/article/pii/S2451958821000890

Thank you for the suggested papers. As discussed in lines 318-328 under the heading "acceptability", beyond the objective acceptability data from the QUEST provided in this paper our acceptability findings will be explored thoroughly in a separate qualitative paper. These suggested papers are very helpful though as we are currently writing the qualitative paper which they are relevant to.

Reviewer: 2
Dr. Maarten Lansberg, THE STANFORD UNIVERSITY MEDICAL CENTER
Comments to the Author:
This is an exceptionally well written paper on a feasibility study of a VR intervention for stroke rehabilitation. The investigators succeeded in recruiting study participants in a very reasonable time (10 months) and they describe the safety and feasibility of using a VR intervention for stroke rehabilitation. I only have a few minor suggestions for further improvement of the manuscript:
Thank you very much for your kind words Dr Lansberg and for your review and comments on our paper.

1) The authors could include an illustration of the VR system (ball and tablet) to provide the reader with a visual of the intervention
We have added an image of the Neurofenix platform in as figure 1.

2. The team found that the vast majority of participants do not reach the target goal of 45 minutes of use 5 days per week. Since the number and duration of sessions was listed as the main feasibility outcome, the conclusions in abstract and in the main text could highlight this finding which is consistent with other interventions aimed at increasing rehabilitation dose.
We have added sentences to both the conclusion in the abstract and in the main body of the text to reflect that use of the platform was variable within the intervention group.
In the abstract this can be seen in lines 102-103 "There was a wide range of engagement with the platform in a cohort of stroke survivors which was varied in age and level of impairment."
And in the main body this can be seen in lines 568-570 "There was a wide range of engagement with the VR platform, however through its use rehabilitation intensity for the UL was increased in a cohort of stroke survivors which was varied in age and level of impairment."
